# Compaction of Cohesive Granular Material: Application to Carbon Paste

**DOI:** 10.3390/ma14040704

**Published:** 2021-02-03

**Authors:** Zahraa Kansoun, Hicham Chaouki, Donald Picard, Julien Lauzon-Gauthier, Houshang Alamdari, Mario Fafard

**Affiliations:** 1 Department of Civil and Water Engineering, NSERC/Alcoa Industrial Research Chair MACE3 and Aluminium Research Centre—REGAL, 1065 Avenue de la Medecine, Laval University, Québec, QC G1V 0A6, Canada; hicham.chaouki@gci.ulaval.ca (H.C.); mario.fafard@ceal.aluquebec.com (M.F.); 2 Eddify Technologies Company, 3425 Rue Pierre-Ardouin, Québec, QC G1P 0B3, Canada; houshang.alamdari@gmn.ulaval.ca; 3 Alcoa Corporation, Continuous Improvement Smelting Technology, 1 Boul. Des Sources, Deschambault-Grondines, QC G0A 1S0, Canada; julien.lauzon-gauthier@alcoa.com; 4 Department of Mining, Metallurgical and Materials Engineering, NSERC/Alcoa Industrial Research Chair MACE3 and Aluminium Research Centre—REGAL, 1065 Avenue de la Medecine, Laval University, Québec, QC G1V 0A6, Canada; donald.picard@gci.ulaval.ca

**Keywords:** carbon paste, compaction, quasi-static behavior, dynamic behavior, cyclic behavior

## Abstract

Carbon-like materials such as the anode and the ramming paste play a crucial role in the efficiency of the Hall–Héroult process. The mechanical behavior of these materials during forming processes is complex and still ill-understood. This work aimed to investigate experimentally the mechanical behavior of a carbon paste used in the aluminum industry under different loading conditions. For this purpose, experiments consisting of (1) relaxation tests at different compaction levels, (2) quasi-static cyclic tests at several amplitudes, (3) monotonic compaction tests at varied strain rates, and (4) vibrocompaction tests at different frequencies were carried out. The obtained results highlight some fundamental aspects of the carbon paste behavior such as the strain rate’s effect on the paste compressibility, the hardening-softening behavior under cyclic loadings, the effect of cycling amplitude on the stress state and the paste densification, and the frequency effect on the vibrocompaction process. These results pave the way for the development of reliable rheological models for the modeling and the numerical simulation of carbon pastes forming processes.

## 1. Introduction

Primary aluminum is produced through the Hall–Héroult process, which involves an electrolytic reduction operation driven by an electrical current. The alumina (Al_2_O_3_) is dissolved in a molten bath of cryolite (Na_3_AlF_6_), and an electrical current flows through the prebaked carbon anodes, which are immersed into the bath. The oxygen, present in the alumina, reacts with the carbon anode leading to the production of carbon dioxide (CO_2_), and the molten aluminum is deposited on the top surface of the carbon cathode, which acts as a collector of the electrical current. The overall reaction is resumed through the following expression:

(1) $$2Al_{2}O_{3}+3C\to 4Al+3CO_{2}$$

The primary aluminum production is an energy-intensive process [1,2,3]. In the best modern practices, the production of 1 kg of aluminum requires an electrical power almost equal to 13 kWh [1,2,3], while the theoretical value is almost 6.34 kWh [4]. Accordingly, great attention must be paid in order to optimize the Hall–Héroult process.

Carbon-like materials such as the anode and the ramming paste are key parts of electrolysis cells, as they play a crucial role in the efficiency of the Hall–Héroult process. For instance, the voltage drop associated with the anode assembly represents approximately 6.5% of the total cell’s voltage drop [5].

Green anodes are composed of carbon aggregates, coal tar pitch, and recycled anode butts. Nowadays, most plants use the vibrocompaction process for anode production. This process allows the production of anodes with higher quality in comparison to the monotonic compaction process [6]. Afterwards, anodes are baked at 1100 °C over a cycle of 12–14 days (preheating, baking, cooling) before being immersed in the electrolysis cell bath of cryolite. The presence of slots and stub holes in the anode forming process leads to non-negligible density gradients which may deteriorate anodes’ quality (e.g., cracks after baking, high electrical resistivity, and anode’s high consumption) [7,8]. Accordingly, some relevant anode physical properties such as electrical resistivity, porosity, and mechanical properties are closely related to the green anode quality. The ramming paste is used as a joint between cathodes and as a peripheral seam in the cell; it ensures the pot tightness and absorbs thermal expansion of cathode blocks [9,10]. It consists of carbon aggregates, coal tar pitch, and softeners that allow a forming process at room temperature, and it is rammed layer by layer. The ramming paste is baked during the cell preheating phase and undergoes some complex phenomena such as swelling and shrinkage. A poorly compacted ramming paste may suffer after the baking from a significant shrinkage and cracks, which may substantially affect the cell life span through metal and the bath infiltration in the electrolysis cell lining [10]. Therefore, controlling and improving the qualities of these two carbon materials is of high interest for aluminum industry. Nevertheless, addressing these challenges using the in-situ trial and error approach involves considerable investments. In this context, the modeling approach seems to be an appropriate alternative. This approach is based, among others, on the experimental characterization of the mechanical behavior, which enables the development of appropriate constitutive laws to predict the material behavior.

In the literature, few research works have been done to characterize the mechanical behavior of carbon-like materials used in the primary aluminum production, especially regarding their mechanical behaviors under cyclic loading. In [11,12], the effects of raw materials, such as particles shape and cock/pitch ratio, on the green anodes mechanical behavior during the monotonic compaction was investigated. In [13], the mechanical behavior of the green anode paste during the compaction process was characterized at 150 °C using monotonic and cyclic compaction tests. Experimental results in the quasi-static regime have shown that a small axial stress level leads to green anode’s significant densification before the skeleton takes form. Afterwards, the stress increases substantially for further paste’s densification. The same trend was also observed for radial behavior. In [14], cyclic compaction tests were carried out on dry coke aggregates at a small strain rate where they highlighted the presence of a combined hardening-softening behavior during each loading cycle. In this context, the authors advanced the hypothesis of aggregates breakage to explain the softening behavior. In [15], a viscoplastic constitutive law was developed to simulate the green anode paste behavior during the monotonic compaction process. In [16], a modified Cam–Clay model was used to characterize the ramming paste behavior. The effects of the vibrocompaction process parameters on anodes quality have been investigated in some experimental works [6,17,18,19,20]. The results obtained have shown that increasing some process parameters, such as the vibration time and the frequency, generally improves the anode properties (e.g., apparent density, electrical resistivity, and mechanical properties) [6,17,19,20]. Nonetheless, the existence of an optimal vibration’s time beyond which the anode’s properties may deteriorate, due to the over-compaction, has been shown in [6,18]. Similar trends have also been observed for the over-compacted ramming paste in [10]. Furthermore, the effect of mixing time of raw materials on the anode properties has been investigated in [17]. A few attempts have been made to develop dynamical models aiming at modeling the anode vibrocompaction process [7,8,21,22]. In such research works, dynamic one-dimensional models were developed using combinations of springs and dashpot elements. Despite the complex mechanical behavior of anode during the densification, only simple mechanical properties, such as the stiffness and Young modulus of the anode paste, were considered, and the rheological behavior of the anode was not taken into account.

From another standpoint, asphalt mixes, which possess a similar composition of the anode paste mixture, have received considerable attention. In this context, several studies have been carried out to investigate the mechanical behavior of hot asphalt mixes during the compaction processes adopting the viscoplasticity theory [23,24,25]. Furthermore, the mechanical behavior of asphalt materials under cyclic loadings has also been investigated to characterize the rutting phenomenon [26,27,28]. In these research works, the mechanical behavior of asphalt mixes under cyclic loads involves some important phenomena such as (1) hardening-relaxation behavior, (2) particles rearrangement, and (3) significantly higher permanent deformation generated by cyclic loads compared to monotonic loads.

It emerges from the literature review that the mechanical behavior of anode and ramming pastes under cyclic loadings or during forming processes are still ill-understood. The present work aims to investigate the mechanical behavior of a carbon paste used in the aluminum industry. For this purpose, an experimental campaign was carried out which consists of (1) relaxation tests at different densities, (2) cyclic compaction tests at different amplitudes, (3) monotonic compaction tests at several strain rates, and (4) vibrocompaction tests at various frequencies. Results obtained highlight some interesting insights into the behavior of carbon pastes such as the hardening-softening behavior associated with cyclic loadings, and the effects of strain rates on the compaction of carbon pastes.

## 2. Methodology

### 2.1. Material and Set-Up

**Figure 1:**
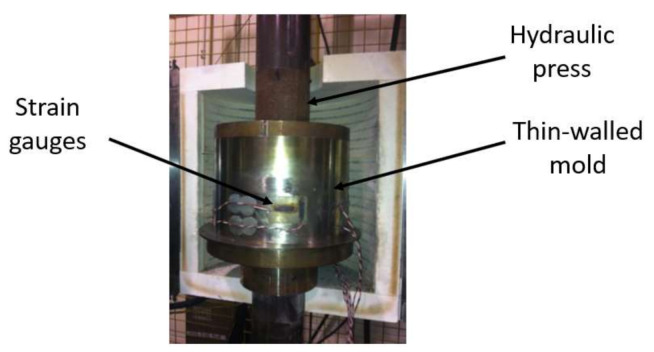
Experimental set up.

A thin-walled mold made of 18/10 stainless-steel was used for all the experimental tests presented in this paper (Figure 1). The mold has an inner diameter of 254 mm, a height of 140 mm, and a uniform thickness of 0.356 mm. The mold was instrumented by 8 strain gauges: 4 axial and 4 radial ones, which were placed in pairs and were equally spaced on the circumference of the mold at a height of 40 mm. The gauges measurements allow the calculation of the radial pressure and the sample radial deformation using the theory of the thin shells detailed in [15]. The mechanical properties of the mold were characterized using a simple tensile test, which were equal to *E* = 220 GPa and *υ* = 0.31. *E* and υ denote the Young’s modulus and the Poisson ratio, respectively. Tests were performed using a DARTEC hydraulic press with a maximum load capacity of 250 kN. The axial load was applied by a computer-controlled servo-hydraulic actuator. The press was modified in the past, the controller is an MTS FlexTest 40 (MTS Systems Corporation, Eden Prairie, MN, USA), and the load cell is a 250 kN MTS (model 661.22H-01, MTS Systems Corporation, MN, USA). The piston of the load cell has a diameter of 250 mm, a gap of 2 mm between the mold on the piston exists to avoid any friction between them. The displacement of the press piston was measured by a calibrated LVDT (Linear variable differential transformer)that has a measurement range of +120 mm and an accuracy of ±0.001 mm.

Performing experiments on the green anode paste (GAP) requires, among other things, a strict control of the temperature since the pitch viscosity is highly temperature sensitive and requires a constant initial density for all tests. These requirements make carrying out such tests a difficult task. To overcome these technical problems, the work presented in this article is done with a room temperature alternative carbon paste (ACP). The ACP is a commercially available room temperature ramming paste used in the electrolysis cell. The ACP is made essentially of carbon aggregates and coal tar pitch. In addition to these raw materials, the ACP contains a softener that reduces the room temperature viscosity of the pitch to be close to its viscosity at high temperature. Due to the confidentiality, the ACP composition recipe will not be disclosed.

Before each test, the mold’s internal wall was coated with a thin layer of a lubricant oil in order to limit the ACP/mold friction. Then, 6 kg of ACP was placed in the mold in a loose state. The initial height of the sample for all tests was $h_{i}=$ 135 mm ± 2 mm.

Four series of experiments were carried out in order to characterize the ACP rheological behavior: (1) relaxation tests at different compaction levels, (2) quasi-static cyclic tests at several amplitudes, (3) monotonic compaction tests at different strain rates, and (4) vibrocompaction tests at different frequencies. In this study, the interest will be focused on the compacted ACP up to a density pertaining to the range of [1.6 g/cm^3^–1.65 g/cm^3^], which is the average green density of industrial anodes [29]. Each test was repeated two times, and all the error bars in the graphs represents the standard deviation of our results.

### 2.2. Relaxation Tests

The first series of experiments involved nine relaxation tests at different imposed strains. The purpose of these tests was to explore the viscous behavior of the ACP and to identify how this behavior would vary as the imposed strain increases. In other words, the effect of the compaction degree (density) on the reversible and dissipative behaviors of the ACP were investigated.

**Figure 2:**
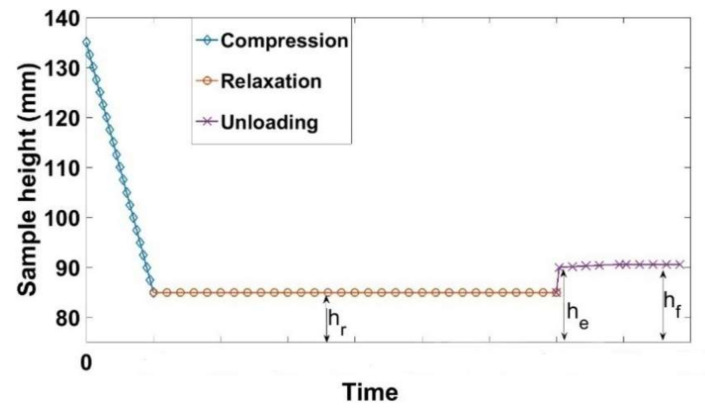
Height of the sample during the relaxation test.

The load path of the relaxation tests is shown in Figure 2. The relaxation test consists firstly of a monotonic compaction phase up to a target sample height of *h_r_* with a strain rate of $\dot{\varepsilon }$ = 0.006 s^−1^. Then, a relaxation phase follows; the sample height *h_r_* is maintained constant for a period of 300 s, and the stress is recorded. Subsequently, the load is removed in the recovery phase, and the paste displacement is recorded until the final height *h_f_* is reached. Therefore, one can characterize the stress relaxation, the instantly recoverable strain (*ε_e_*), the time-dependent strain (*ε_a_*), and the permanent strain (*ε_p_*). The densities of the samples during the relaxation phase are constant since the sample height (*h_r_*) is constant, their values for the nine tests are depicted in Table 1.

### 2.3. Quasi-Static Cyclic Tests

This series of experiments comprises four cyclic compaction tests with increasing values of maximum amplitudes and one monotonic compaction test. They were driven all (including the monotonic compaction test) at the same strain rate of $\dot{\varepsilon }$ = 0.006 s^−1^. The aim of these experiments is to highlight the differences between monotonic and cyclic loadings and to investigate the effects of the amplitude of cyclic loadings on the ACP compaction behavior.

**Figure 3:**
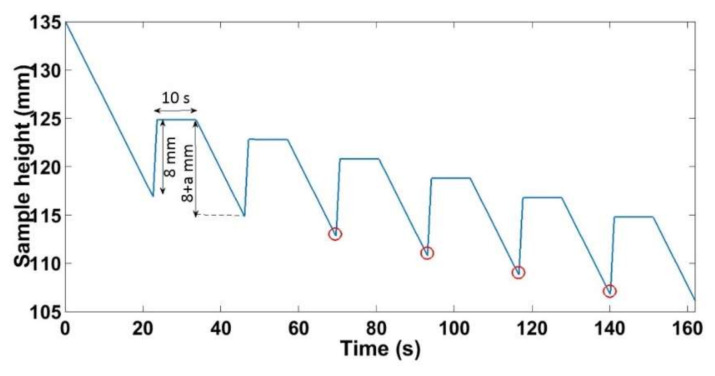
Loading path of the quasi-static cyclic compaction test.

For the quasi-static cyclic tests, the paste was compacted in a quasi-static regime according to the load path shown in Figure 3. In a first step the sample was compacted monotonically from an initial height of *h_i_* = 135 ± 2 mm until it reaches a height of *h* = 100 mm. At this stage, the cycling begins by displacing the press 8 mm upward. This value was chosen to ensure that the unloading displacement is greater than the recoverable deformation of the ACP. The sample is kept in this configuration for 10 s. Afterwards, the sample is loaded, and the press is displaced (8 + a) mm downward, where (a) is a prescribed value (Figure 3). This cycle is repeated until a density nearly equal to 1.65 g/cm^3^ is reached. Tests were performed for a = 0.25 mm, a = 0.5 mm, a = 1 mm, and a = 2 mm. The red circles in Figure 3 represents the points where the stress is maximal during each cycle.

### 2.4. Monotonic Compaction Test at Different Strain Rates

The aim of these tests is to investigate the difference between the static and the dynamic behaviors of the ACP. To this end, three quasi-static monotonic compaction tests were conducted at strain rates of: 0.0074 s^−1^, 0.0222 s^−1^, and 0.0370 s^−1^. Two more dynamic monotonic tests were conducted at strain rates of: 0.3703 s^−1^ and 0.7407 s^−1^. The compaction of the latter two tests took less than two seconds.

### 2.5. Vibrocompaction Tests

**Figure 4:**
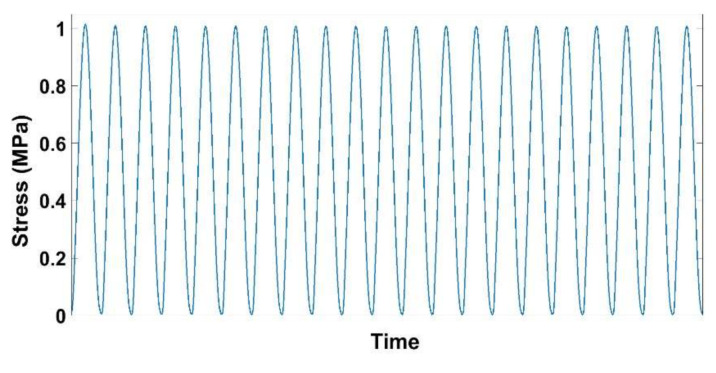
Stress variation during vibrocompaction tests.

These tests aim at investigating the effect of the vibrocompaction frequency on densification and the rigidity of the vibrocompacted samples. To this end, vibrocompaction tests were carried out at the following frequencies: 0.1 Hz, 2 Hz, 4 Hz, and 7 Hz. Tests with higher frequencies that approach the industrial frequency of vibrocompaction (≈25 Hz) could not be carried out with the hydraulic press used in this work. The samples were subjected to a sinusoidal stress that had a maximal amplitude of 1 MPa (Figure 4). The vibration stopped when the density of the sample reached 1.65 g/cm^3^. After the vibrocompaction, a rigidity test was performed on some of the compacted samples. This test consists in loading the molded sample with a deformation rate of $\dot{\varepsilon }$ = 0.006 s^−1^ until reaching an axial stress of 1 MPa. An unloading follows and the recoverable deformation is recorded at the end of the unloading. The rigidity modulus is then calculated by dividing the maximum stress of loading by the corresponding recoverable deformation.

## 3. Results and Discussion

### 3.1. Relaxation Tests

The results of the relaxation tests conducted according to the load path shown in Figure 2 are described and analyzed in this section. The measured data during each test are: (1) the stress during the relaxation phase and (2) the height of the sample. The stress during the relaxation phase (*σ*(*t*)) is normalized with respect to the maximum stress reached during this phase (*σ_n_* = *σ*(*t*)/*σ_max_*), which is the initial value of the relaxation stress.

**Figure 5:**
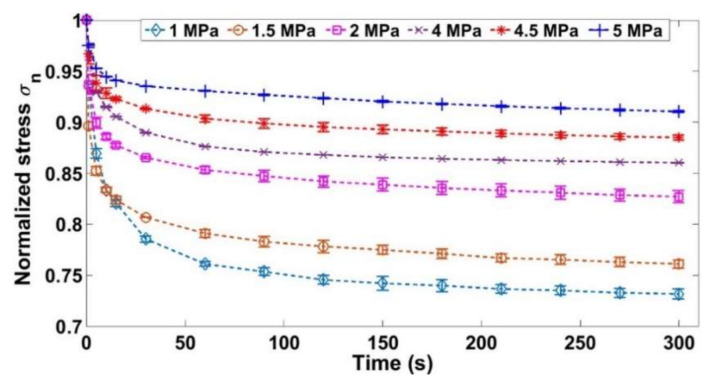
Normalized stress for the relaxation tests.

Figure 5 shows the evolution of the normalized stress as a function of the time for only six tests out of nine for the sake of clarity. The densities for the relaxation phases and their corresponding maximum stresses are shown in Table 2. Note that the radial deformation of the samples varies during the relaxation phase. However, this variation is negligible compared to the initial mold’s radius. Thus, it does not affect the density estimation, which can be considered constant during the relaxation phase.

During the tests where the relaxation density is low, the normalized stress drops more considerably compared to the tests where the density during relaxation is higher (Figure 5, Table 2). At low densities, the stress drop could be explained by the fact that the aggregates will rearrange themselves (keeping a constant volume) in a way to reduce the stress during relaxation. As the time passes, the aggregates become less constrained in their new configuration (decrease of contact forces). For higher densities (less stress drop of the normalized stress), there is not enough free space for the aggregates to rearrange since the skeleton is consolidated [30].

**Figure 6:**
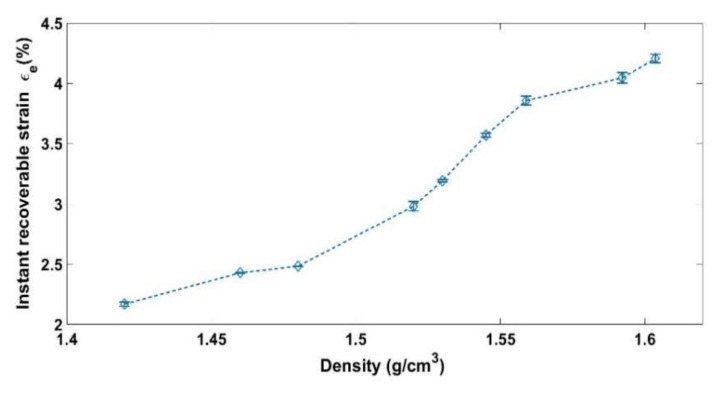
Instantly recoverable strain as a function of the relaxation density.

**Figure 7:**
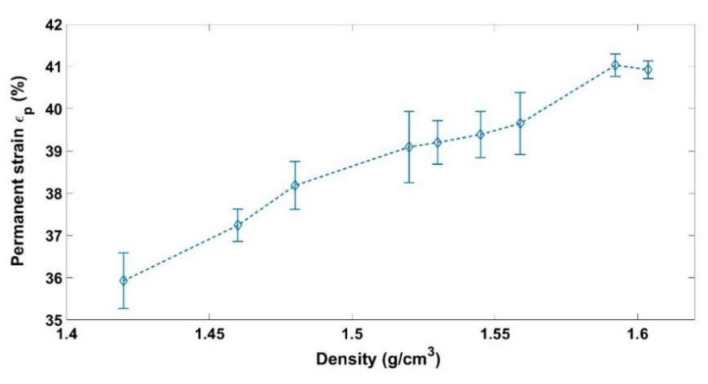
Permanent strain as a function of the relaxation density.

**Figure 8:**
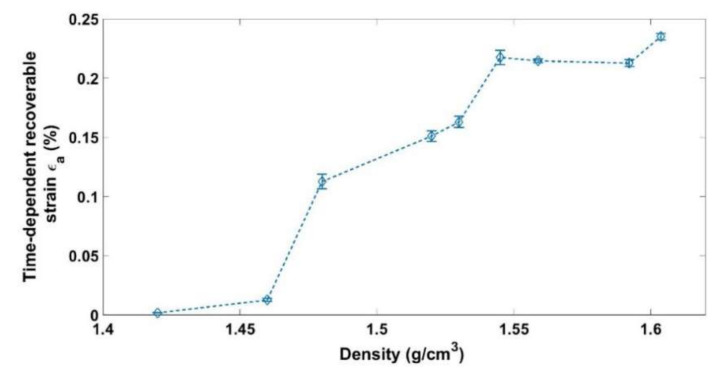
Time dependent recoverable strain as a function of the relaxation density.

At the end of the relaxation phase, the load is removed. Therefore, the sample height increases instantly from *h_r_* to *h_e_*. Afterwards, it continues to increase slowly to reach a constant height (*h_f_*) after a certain time. The evolution of the sample height during the relaxation and the unloading phases (Figure 2) will be used to calculate the instant recoverable strain ($\varepsilon_{e}$), the time-dependent recoverable strain ($\varepsilon_{a}$), and the permanent strain ($\varepsilon_{p}$) at the end of the relaxation phase of each test according to the following equations:

(2) $$\varepsilon_{e}=\frac{h_{e}-h_{r}}{h_{i}}$$

(3) $$\varepsilon_{a}=\frac{h_{f}-h_{e}}{h_{i}}$$

(4) $$\varepsilon_{p}=\frac{h_{i}-h_{f}}{h_{i}}$$

where $h_{i}$ represents the initial height of the sample. Figure 6, Figure 7 and Figure 8 show respectively the values of the instantly recoverable, permanent, and time-dependent strains at the end of relaxation phase for the nine relaxation tests. For each test, these strains are plotted in function of the sample apparent density during the relaxation phase. According to the instant recoverable strain rate evolution, two phases are distinguished:

Phase 1 $\left[\rho \approx 1.42\frac{g}{{\mathrm{cm}}^{3}}-\;\rho \approx 1.48\;g/{\mathrm{cm}}^{3}\right]:$ The elastic strain evolution in function of the density is small in comparison with the next phase. This could be an indication that the solid skeleton is not yet consolidated and that the permanent deformation is due to the evacuation of the air from the sample and the particle rearrangement.

Phase 2 [$\rho \approx 1.48\;\frac{g}{{\mathrm{cm}}^{3}}-\rho \approx 1.6\;g/{\mathrm{cm}}^{3}]:$ The elastic strain evolution in function of the density becomes more important with respect to the previous phase. This could be an indication of the increase in the contact forces between the aggregates and the solid skeleton consolidation.

The time-dependent strains measured for the nine tests are all negligible compared to the instantly recoverable and permanent strains (Figure 8). However, this strain starts to evolve significantly during the second phase where the solid skeleton is formed.

### 3.2. Quasi-Static Cyclic Tests

**Figure 9:**
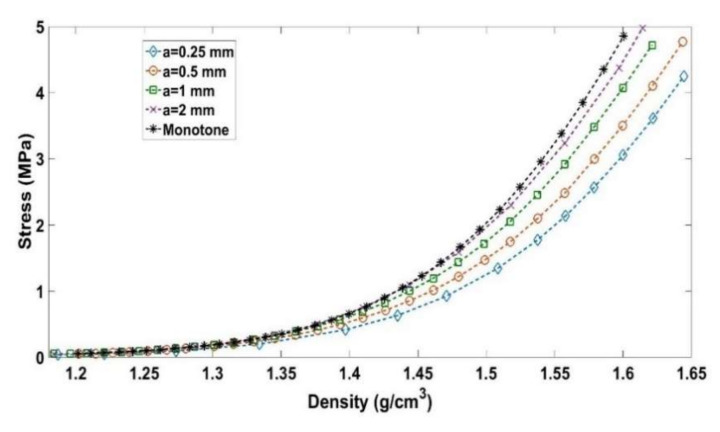
Stress evolution as a function of the density: cyclic vs. monotonic compaction tests.

Quasi-static cyclic tests at different loading amplitudes (a = 0.25 mm, a = 0.5 mm, a = 1 mm, and a = 2 mm) were performed at the same strain rate of $\dot{\varepsilon }$ = 0.006 s^−1^, according to the load path shown in Figure 3. Moreover, a monotonic compaction test was carried out to compare its results to those of the cyclic tests. Figure 9 depicts the evolution of the stress as a function of the density for all tests. For the cyclic tests, the density and the stress plotted in Figure 9 correspond to the encircled points of Figure 3.

One can notice that samples compacted using cyclic loading require less stress than the monotonic compaction test does to reach a defined density. Furthermore, it seems that decreasing the amplitude of cyclic loading significantly reduces the required stress level to reach the target density. Table 3 summarizes stress levels needed to obtain a target density of $\rho =1.6\;g/{\mathrm{cm}}^{3}$. Compared to the monotonic compaction test, cyclic loading tests lead to a stress reduction of 9.9% for a = 2 mm, 16.2% for a = 1 mm, 28% for a = 0.5 mm, and 37.24% for a = 0.25 mm.

**Figure 10:**
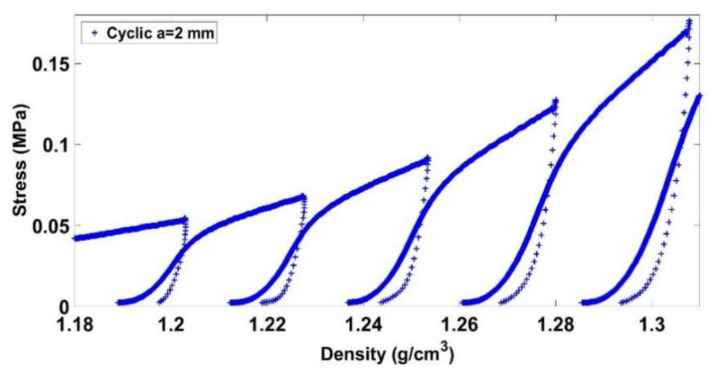
Stress vs. density $\left(1.18\;g/{\mathrm{cm}}^{3}\leq \rho \leq 1.35\;g/{\mathrm{cm}}^{3}\right)$.

**Figure 11:**
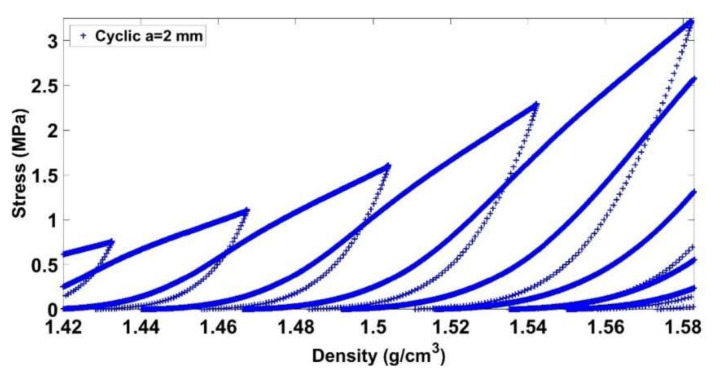
Stress vs. density $\left(1.42\;g/{\mathrm{cm}}^{3}\leq \rho \leq 1.58\;g/{\mathrm{cm}}^{3}\right)$.

Before giving an explanation on this trend, let us analyze the mechanical behavior of the carbon paste under a cyclic loading compaction test. Figure 10 and Figure 11 illustrate the stress evolution for the cyclic test with a = 2 mm at low and high densities, respectively. For a lower density, the paste is initially compacted with a small stress level. A hardening behavior is subsequently observed. Once the maximum density of the previous loading cycle is reached, we observe a softening behavior highlighted by a change of the curve’s slope. For higher densities (Figure 11), similar trends are observed. However, the softening behavior is less noticeable with higher densities.

This behavior has been reported in some research work dealing with geomaterials and the anode paste [14]. In [14], cyclic compaction tests were carried out on dry petroleum coke aggregates. In this context, an experimental procedure based on sieving dry aggregates after each loading cycle and the measurement of aggregates’ acoustical emissions has shown that this softening behavior is due to the aggregates’ crushing and slipping. Once the maximum density of the previous cycle is reached, aggregates start to crush and slip [14].

**Figure 12:**
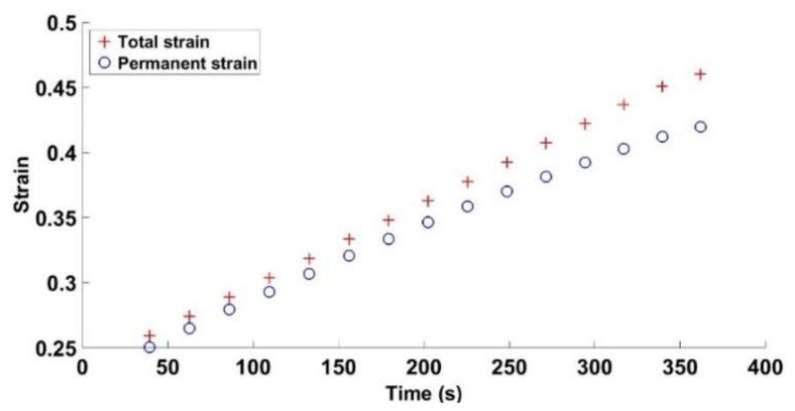
Total and permanent strains vs. time for the cyclic compaction test with a = 2 mm.

**Figure 13:**
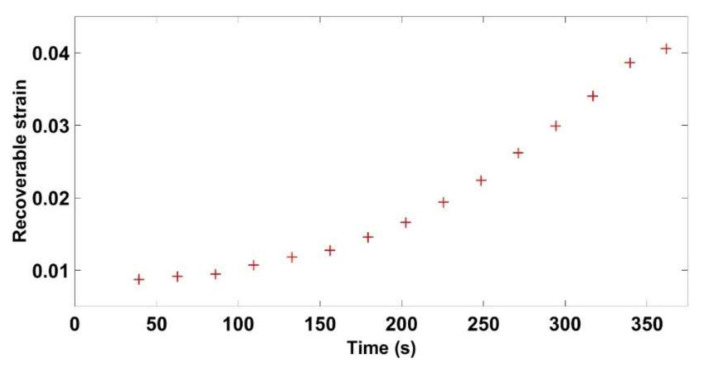
Recoverable strain vs. time for the cyclic compaction test with a = 2 mm.

**Figure 14:**
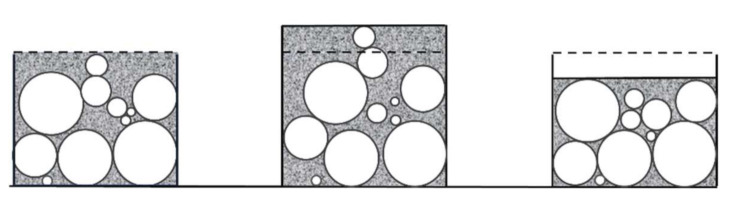
Rearrangement of particles during cyclic loading. From left to right: loading, unloading, and next loading. Dashed line: level after the initial compaction, solid line: current phase level.

Figure 12 and Figure 13 depict the evolution of total, permanent, and recoverable strains as a function of time for the cyclic test with a = 2 mm. The total strain is calculated at the points encircled in Figure 3, while the permanent and recoverable strains are estimated at the end of unloading phases of each cycle. At the first compaction stages, the carbon paste is in a loose state. Thus, after an unloading phase, the paste recovery is almost negligible, and the recoverable strain is small. As the density increases, the skeleton takes form, and the strain recovery starts to evolve leading to the increase in the difference between the total strain of the paste and its permanent strain. During a loading phase, the binder matrix is under compression as is the air pores. This compression generates a stress state that relaxes during the unloading phase and leads to particles rearrangement in the next cycle as schematically depicted in Figure 14. Therefore, in the subsequent loading phase, one can reach the maximum density of the previous cycle with a lower stress level (Figure 10 and Figure 11). In [27], the effect of cyclic loading on the behavior of compacted asphalt mixes was investigated. The authors emphasized the effect of unloading phases on particles rearrangement in terms of leading to better densification.

**Figure 15:**
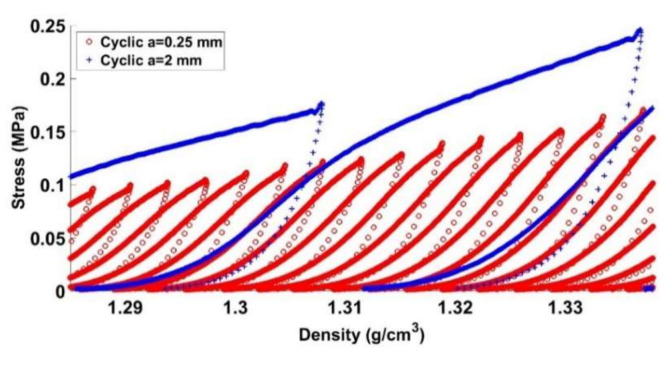
Stress vs. density: cyclic tests with a = 0.25 mm and a = 2 mm.

**Figure 16:**
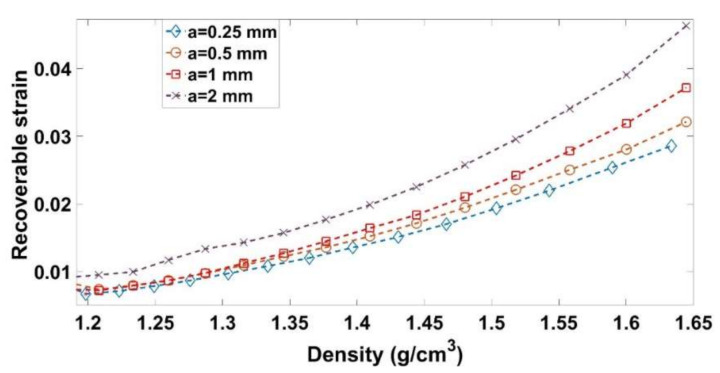
Recoverable strain vs. density for cyclic tests.

Figure 9 highlights the effect of the amplitude on the stress level. For a given density, the smaller the amplitude is, the lower the stress will be. Moreover, for all amplitude levels, a softening behavior is observed once the maximum density of the previous cycle is reached. Let us consider a cycle C_1_ corresponding to the amplitude a_1_ = 2 mm and covering a density range [$\rho_{1}-\rho_{1}+\Delta \rho_{1}$]. For a smaller amplitude of a_2_ = 0.25 mm, several cycles are needed to cover the same density range (Figure 15). For each cycle, the loading phase generates a pore pressure and internal stress in the binder matrix, while the unloading phase leads to the relaxation of these stresses and therefore to particles rearrangement that enhances the carbon paste compaction during subsequent cycles. Accordingly, for the smaller amplitude of a_2_, each cycle will generate a smaller pore pressure and stress inside the binder matrix, which are relaxed during the unloading phases. Therefore, to cover this density range, the material goes through several intermediate unloaded configurations allowing a better particles rearrangement than the cyclic test with the amplitude a_1_, where higher pore pressure and stress inside the binder matrix are generated. Furthermore, these higher internal stress states are expected to lead to a higher recoverable strain. Figure 16 depicts the evolution of the recoverable strain as a function of the density for all cyclic tests. The higher the amplitude is, the higher the recoverable strains will be. Therefore, at the same density, the sample compacted with a lower amplitude will have a higher permanent deformation (compaction degree) than the sample compacted at a higher amplitude.

### 3.3. Monotonic Compaction Tests at Different Strain Rates

**Figure 17:**
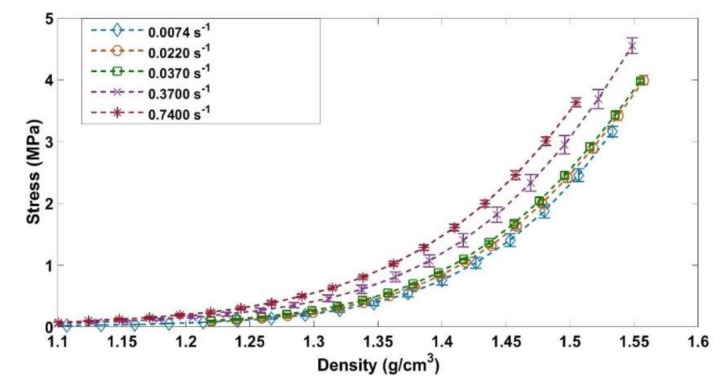
Effect of the strain rate on the compaction behavior.

Figure 17 shows the stress evolution as a function of the density for the ACP for strain rates corresponding to 0.0074 s^−1^, 0.0222 s^−1^, 0.0370 s^−1^, and 0.7407 s^−1^. The curves that represent the quasi-static compaction (0.0074 s^−1^, 0.0222 s^−1^, and 0.0370 s^−1^) are superimposed. There is no effect of the strain rate in the quasi-static regime. This result was noted in [31] for the GAP. For compaction tests carried out in the dynamic regime (0.3703 s^−1^ and 0.7407 s^−1^), one can notice that the paste compressibility is dependent on the strain rate. The increase of the strain rate leads to an increase in the stress needed to reach the target density. Considering that the compaction in this regime takes less than 2 s, one can assume that for higher strain rates the air does not have enough time to escape from the ACP. This entrapped air generates a pore pressure that contributes to the increase of the uniaxial stress. Thus, to increase the compressibility of the ACP during monotonic compactions, low strain rates should be adopted.

### 3.4. Vibrocompaction Tests

A series of vibrocompaction tests were carried out in order to investigate the effect of the frequency on the ACP densification under vibrations. The target density for all these tests was 1.65 g/cm^3^. Recall that the applied load consists of a sinusoidal stress ranging between 0 and 1 MPa (Figure 4).

**Figure 18:**
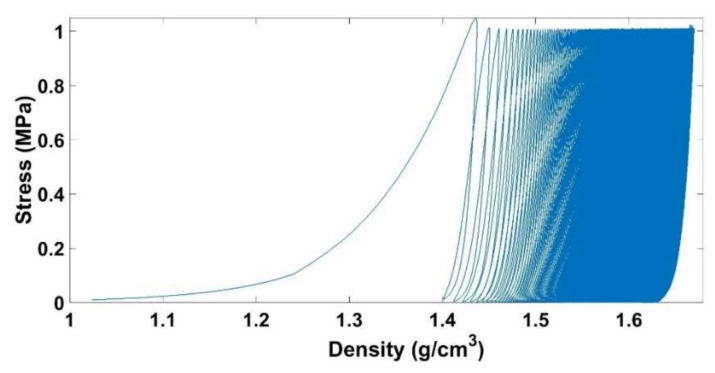
Stress vs. density for the vibrocompaction test with a frequency of f = 0.1 Hz.

It is notable that the vibrocompaction requires a small stress of 1 MPa to densify the ACP to a density of 1.65 g/cm^3^ while a stress of 4.9 MPa is needed in the monotonic compaction (Figure 9). Figure 18 shows the stress in function of the density for a vibrocompaction test with a frequency of f = 0.1 Hz. The evolution of the density per cycle is higher when the density is lower, and it decreases gradually when the density increases.

**Figure 19:**
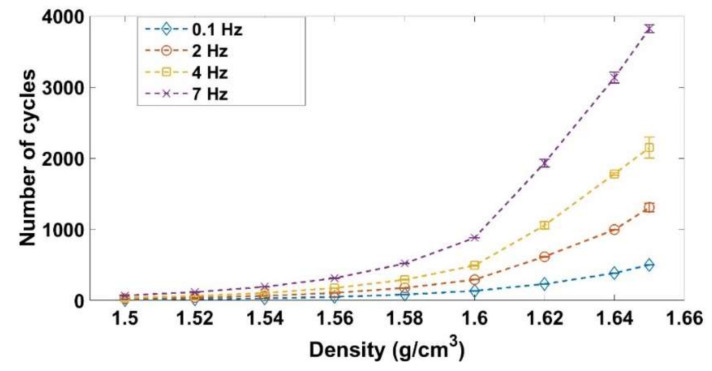
Density in function of the number of cycles during the vibrocompaction for frequency = 0.1 Hz, 2 Hz, 4 Hz, and 7 Hz.

**Figure 20:**
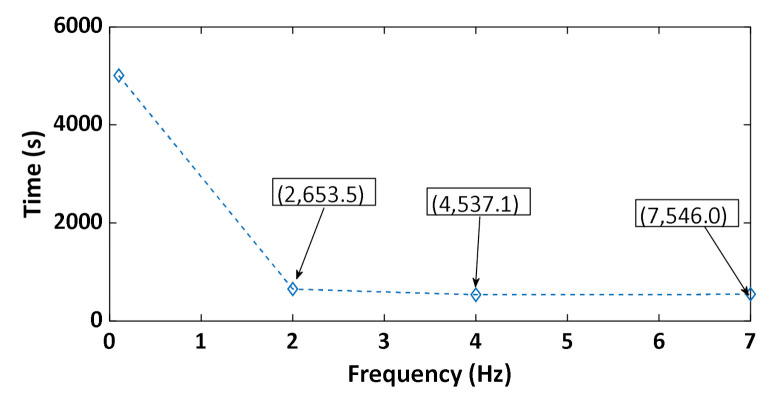
Total time of vibrocompaction as a function of the frequency (the numbers in the brackets represent the points coordinates).

Figure 19 shows that increasing the frequency leads to an increase in the number of cycles needed to reach the target density. Increasing the frequency has two effects. On the one hand, to increase the frequency means to increase the strain rate during the loadings, which decreases the capacity of the material to be compacted (Section 3.3). On the other hand, increasing the frequency means decreasing the duration of unloading when the pressure of the air is supposed to be relaxed. The time needed to reach a density of 1.65 g/cm^3^ under vibrocompaction for different frequencies is shown in Figure 20. Increasing the frequency from 0.1 Hz to 2 Hz decreases the vibrocompaction time considerably. However, there is no considerable change in the vibrocompaction time between the tests run at 2 Hz, 4 Hz, and 7 Hz.

Table 4 shows the rigidity of the vibrocompacted samples. As mentioned, all samples are compacted to a density of 1.65 g/cm^3^ and under a maximum stress amplitude of 1 MPa. The results show that the frequency has no effect on the rigidity of the samples for the studied frequencies, if they are compacted to the same density and under the same maximum amplitude of stress.

## 4. Conclusions

This paper aimed at characterizing the mechanical behavior of a commercial-grade carbon paste used in the aluminum industry. To this end, an experimental campaign consisting of relaxation tests, cyclic and monotonic compaction tests, and vibrocompaction tests was carried out. The obtained results are summarized as follows:

Relaxation test: The carbon paste undergoes a significant stress relaxation for small densities. This relaxation becomes less important when the carbon paste’s density increases. The analysis of the strain components highlighted the existence of two main phases. During the first phase ($\rho <1.48\;g/{\mathrm{cm}}^{3}$), the permanent strain evolved significantly compared to the instantly reversible strain. The opposite behavior was observed in the second phase, where $\rho >1.48\;g/{\mathrm{cm}}^{3}$. These trends can be explained by the solid skeleton formation. Once the skeleton is formed, contact forces between aggregates become significant. Consequently, the viscous behavior and the permanent strain evolve less significantly in contrast to the instantly reversible strain, which becomes higher.

Quasi-static cyclic tests: The compaction using cyclic loading tests requires less stress than the monotonic one. Moreover, decreasing the amplitude of the cyclic loading leads to a further decrease in the stress needed to densify the paste. This behavior can be explained through particles’ rearrangement and the relaxation of stress in pores induced by unloading phases. Cyclic loading tests showed that the maximum density of a given cycle is reached in the subsequent cycle with a lower stress state.

Monotonic compaction tests at different strain rates: In the quasi-static regime, the strain rate does not affect the paste’s behavior. However, in the dynamic regime when the strain rate increases the paste compressibility decreases, and more stress is needed to reach the target density. At high strain rates, the time of compaction is very short and the air entrapped in the paste does not have enough time to escape. Accordingly, significant pore pressure is generated, which resists against the compaction of the paste.

Vibrocompaction tests: By increasing the frequency of vibrocompaction tests the number of cycles needed to reach the target density increases significantly while the time required to obtain the target density decreases. Furthermore, tested frequencies seem to not affect the rigidity of vibrocompacted samples.

These results bring new insights related to the mechanical behavior of carbon pastes subjected to a variety of loading conditions. They pave the way for a better understanding of the forming processes of this class of materials and for the development of relevant constitutive laws enabling the modeling of the behavior of carbon pastes. Nevertheless, more investigations are required on the ACP behavior. Especially, the effect of the maximum stress amplitude, the strain rate effect during cyclic loading, and the effect of the residual stress at the end of the unloading phases.

## Figures and Tables

**Table 1:** Density of the sample during the relaxation phases.

Test	1	2	3	4	5	6	7	8	9
Density (g/cm^3^)	1.42	1.46	1.48	1.52	1.53	1.55	1.56	1.59	1.60

**Table 2:** Density and maximum stress during the relaxation phases of relaxation tests.

Test	1	2	3	4	5	6	7	8	9
Relaxation density (g/cm^3^)	1.42	1.46	1.48	1.52	1.53	1.55	1.56	1.59	1.6
Maximum stress (MPa)	1	1.5	2	2.5	3	3.5	4	4.5	5

**Table 3:** The maximum stress needed to obtain a target density of $1.6\;g/{\mathrm{cm}}^{3}.$

Test		Stress [MPa]
Monotonic		4.86
Cyclic	a = 2 mm	4.38
	a = 1 mm	4.07
	a = 0.5 mm	3.50
	a = 0.25 mm	3.05

**Table 4:** Rigidity of the vibrocompacted samples at 0.1 Hz, 2 Hz, and 4 Hz.

Frequency (Hz)	Rigidity (MPa)	Standard Deviation (MPa)
0.1	53.78	0.705
2.0	53.23	1.274
4.0	52.45	0.686

## Data Availability

Not applicable.
